# Evaluation of bone marrow-derived mesenchymal stem cell quality from patients with congenital pseudoarthrosis of the tibia

**DOI:** 10.1186/s13018-018-0977-9

**Published:** 2018-10-23

**Authors:** Ismail Hadisoebroto Dilogo, Fajar Mujadid, Retno Wahyu Nurhayati, Aryadi Kurniawan

**Affiliations:** 1 Integrated Service Unit of Stem Cell Medical Technology, Dr. Cipto Mangunkusumo General Hospital (RSCM), Jl. Diponegoro No 71, Salemba, Cental Jakarta, 10430 Indonesia; 2 Stem Cell and Tissue Engineering Cluster, Indonesian Medical Education and Research Institute (IMERI), Faculty of Medicine, Universitas Indonesia, Jl. Salemba Raya No 6, Salemba, Cental Jakarta, 10430 Indonesia; 3 Department of Orthopaedic and Traumatology, Faculty of Medicine, Universitas Indonesia - Dr. Cipto Mangunkusumo General Hospital, Jl. Diponegoro No 71, Salemba, Cental Jakarta, 10430 Indonesia; 4 Department of Biochemistry and Molecular Biology, Faculty of Medicine, Universitas Indonesia, Jl. Salemba Raya No. 6, Central Jakarta, 10430 Indonesia

**Keywords:** Pseudoarthrosis, Mesenchymal stem cells, Osteocytes, Cell differentiation

## Abstract

**Background:**

The treatment of congenital pseudoarthrosis of the tibia (CPT) remains challenging in pediatric orthopedics due to the difficulties in bone union, continuous angulation, joint stiffness, and severe limb length discrepancy. Mesenchymal stem cells (MSCs) therapy offers a complementary approach to improve the conventional surgical treatments. Although the autologous MSC treatment shows a promising strategy to promote bone healing in CPT patients, the quality of MSCs from CPT patients has not been well studied. The purpose of this study is to investigate the quality of MSCs isolated from patients with CPT.

**Methods:**

The bone marrow-derived MSCs from the fracture site and iliac crest of six CPT patients were isolated and compared. The cumulative population doubling level (cPDL), phenotype characteristics, and trilineage differentiation potency were observed to assess the quality of both MSCs.

**Results:**

There were no significant differences of the MSCs derived from the fracture site and the MSCs from the iliac crest of the subjects, in terms of cPDL, phenotype characteristics, and trilineage differentiation potency (all *p* > 0.05). However, MSCs from the fracture site had a higher senescence tendency than those from the iliac crest.

**Conclusion:**

MSC quality is not the main reason for delayed bone regeneration in those with CPT. Thus, autologous MSC is a promising source for treating CPT patients

## Backgrounds

Congenital pseudoarthrosis of the tibia (CPT) is a rare disorder indicated by non-union or false joint, tibial bowing, reduced growth in distal tibial epiphysis, and shortening of the tibia [1], affecting at least 1 in 250,000 people [2]. The clinical manifestations of this condition often appear within the first year of life; however, in some cases, the symptoms develop after reaching the adolescence [3]. The main cause of CPT remains unclear; however, about 40–80% of incidence is related to genetic mutation of *NF1* gene, resulting in dysregulation of a multifunctional protein termed as neurofibromin [4–6].

The treatment of CPT remains challenging in pediatric orthopedics due to the difficulties in bone union, continuous angulation, joint stiffness, and severe limb length discrepancy [7]. Amputation often becomes the only choice when repeated surgeries resulted in failure or worst condition in CPT patients. Mesenchymal stem cell (MSC) therapy offers a complementary approach to improve the conventional surgical treatments [8, 9]. Although the autologous MSC treatment shows a promising strategy to promote bone healing in CPT patients, the quality of MSCs from CPT patients has not been well studied.

Although various protocols for isolating human MSCs from different sources exist, minimal criteria have been concluded to be a standard consensus to identify cells as MSCs [10]. The Mesenchymal and Tissue Stem Cell Committee of the International Society for Cellular Therapy proposes that at least three characteristics are required to define human MSCs, first: become plastic-adherent when cultured in standard conditions; second: express CD105, CD73, and CD90, and lack expression of CD45, CD34, CD14, or CD11b, CD79a, or CD19 and HLA-DR surface proteins; and third: can differentiate into osteogenic, adipogenic and chondrogenic lineages (trilineage differentiation) [11].

In the current study, we evaluated and compared the characteristics of MSCs isolated from the iliac crest and fracture site of the tibia from six CPT patients. The phenotypic characteristics and the cumulative population doubling time (cPDL) from both sources were analyzed to assess the trilineage differentiation and proliferation capacities of MSCs from the CPT patients. Finally, proliferation of MSCs from the CPT patients was compared with MSCs from healthy donors for clarifying if MSC quality is the reason for delayed bone regeneration in the CPT patients.

## Methods

### Subjects

Six patients involved in this study were diagnosed with CPT. The patients have been tested for HIV types 1 and 2, HBV, HCV, syphilis, and TORCH prior to the study. Ages of the patients were 15 years or younger. The bone marrow was aspirated from the iliac crest and the fracture site of the tibia from these CPT patients. For healthy (non-CPT) subjects, six participants (age 20–50 years old) were medically examined and showed no symptoms of CPT. MSCs from these healthy subjects were collected from bone marrow of iliac crest.

### Isolation of bone marrow-derived MSCs [12]

Ten milliliters of bone marrow aspirates were diluted by 10 ml complete medium containing ɑ-MEM (Life Technologies, USA) with 10% of platelet lysate (Indonesian Red Cross, Indonesia), 10 IU/ml of heparin sodium (Pratapa Nirmala, Indonesia), 2 mM of GlutaMAX, 100 units/ml of penicillin G sodium, 100 μg/ml of streptomycin sulfate, and 2.5 μg/ml of amphotericin (Life Technologies). Samples were centrifuged at 400×*g* for 10 min. The supernatant was discarded, and the pellet was diluted by an equal volume of complete medium. Fifteen milliliters of diluted cells were transferred into 75-cm^2^ T-Flask and incubated at 37 °C in normoxia condition. Cells were harvested after 80–90% confluent and then sub-cultured until fifth passage. Viability and the number of cells were analyzed by a dye-excluding method with trypan blue [13–15]. The cPDL was calculated based on the following formula:

$$ \mathrm{cPDL}=3.32\ \left(\log\ N-\log\ {N}_0\right)+X $$

where *N* = final cell number (cells/mL), *N*_0_ = initial cell number (cells/mL), and *X* = initial population doubling level.

### MSC phenotypic characterization

MSCs were harvested after the fifth passage. Cells were treated with trypsin for 5 min at 37 °C to detach the adherent cells. After being washed with phosphate-buffered saline, the cells (2 × 10^5^) were stained with human MSC analysis kit (BD Biosciences, USA) according to the company instruction. Fluorescence antibody cocktails contained positive markers (CD73, CD90, and CD105) and negative markers/NEG (CD34, CD11b, CD19, CD45, and HLA-DR). The stained cells were subsequently loaded into a flow cytometer (FACSCalibur; BD Biosciences)

### Senescence assay

A cellular senescence test was performed by a Senescence Cells Histochemical Staining Kit from Sigma-Aldrich (USA) at the fifth passage according to the manufacturer’s protocol. Percentage of senescent cell was observed under an inverted microscope with ×100 magnification in five fields of view and analyzed by ImageJ 1.50i software (National Institute of Health, USA) [16].

### Differentiation assay

Differentiation assay was conducted to confirm the MSC plasticity. At the fifth passage, cultured cells were harvested and transferred to specific inducing media for chondrogenic, osteogenic, and adipogenic differentiations. The cells were cultured in complete medium to induce spontaneous chondrogenic differentiation. Osteogenic and adipogenic potencies were evaluated by culturing the cells in StemPro Osteogenesis and Adipocyte Differentiation Kits (ThermoFisher Scientific, USA), respectively. All cultures were incubated at 37 °C under a normoxia condition. The induced cells were analyzed after 7, 14, and 21 days of culturing.

For the differentiation assays, the cells were stained with 1% of alcian blue, 2% of alizarin red, and 1.4% oil red O for evaluating their capacity to undergo chondrogenesis, osteogenesis, and adipogenesis, respectively. Percentage of staining area was observed in five fields of view with 100-fold magnification. Differentiation potential was measured as percentage of stained area by ImageJ 1.50i software [17, 18].

### Statistics

SPSS 15.0 software was used to analyze the significant difference between MSCs from the iliac crest bone marrow and fracture site bone marrow, in terms of cPDL, percentage of senescent cells, phenotype characteristics, and differentiation potencies. Numerical data was analyzed using independent *t* test or Mann-Whitney test.

## Results

**Fig. 1:**
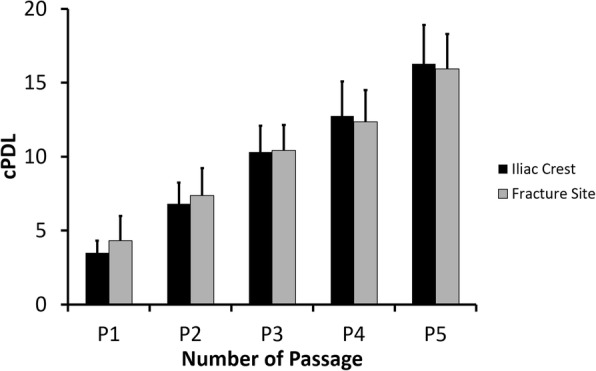
The cPDL of bone marrow-derived MSCs from CPT patients. Data were shown as means, and the vertical bars indicated standard deviations (*n* = 6)

Bone marrow-derived cells gradually attached in plastic surface when cultured in the presence of serum. The adherent cells were subsequently used for further analyses in this study. Afterwards, the cPDL was evaluated to compare the proliferation capacity of bone marrow-derived MSCs from the fracture site and iliac crest of CPT patients. The cPDL from both sources increased from the first passage (P1) to the fifth passage (P5) (Fig. 1), indicating that the MSCs were actively proliferating. In the same passage number, there was no significant difference (*p* > 0.05) of cPDL from MSCs isolated from the iliac crest and fracture site of those with CPT.

**Fig. 2:**
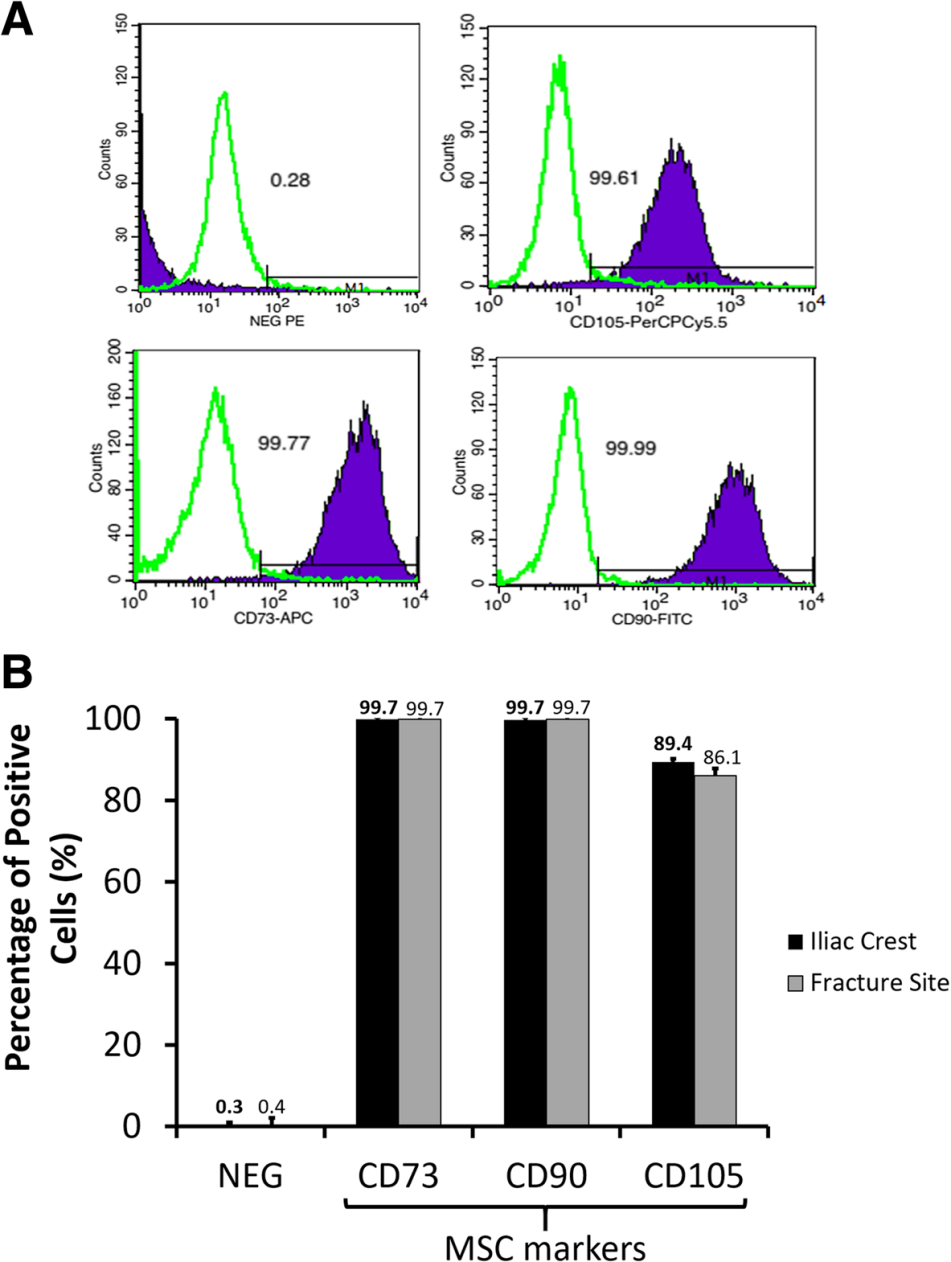
MSC purity of the fifth passaged culture cells isolated from the iliac crest and fracture site of CPT patients. **a** Typical flow cytometer histograms of positive markers (CD105, CD73, CD90) and cocktail of negative markers (NEG) for MSC characterization. **b** Percentage of MSC and non-MSC positive cells. Data were shown as means, and the vertical bars indicated standard deviations (*n* = 6)

After the fifth passage, we evaluated the purity of cultured cells by flow cytometry analysis. More than 99% cultured cells were positive for CD73/CD90, and over 85% were CD105 positive (Fig. 2). Non-MSCs including cells marked positive with CD34/CD11b/CD19/CD45/HLA-DR were less than 0.5%. These results suggested that up to the fifth passage, the iliac crest- and fracture site-derived cells maintained the MSC phenotypes.

**Fig. 3:**
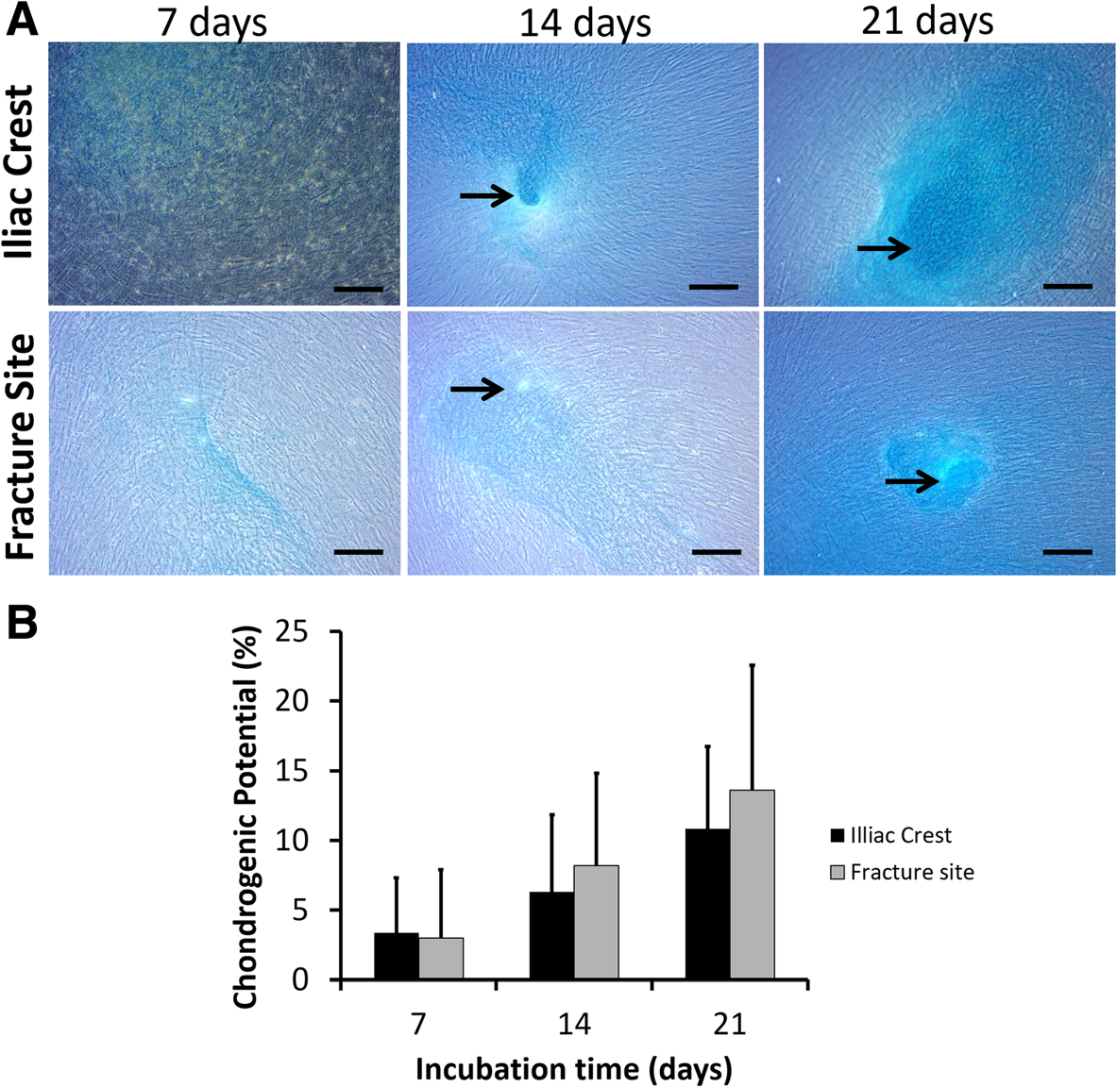
Chondrogenic differentiation of MSCs isolated from the iliac crest and fracture site of the tibia from CPT patients. **a** Representative microscopic images of chondrogenic assays. Bars and black arrows indicate 100 μm and chondrocytes population, respectively. **b** Percentage of chondrogenic differentiation potential**.** Data were shown as means, and the vertical bars indicated standard deviations (*n* = 6)

Figure 3 showed the positive result of chondrogenic differentiation assay as the blue-stained area. Chondrocyte population appeared on day 14 after incubation. Both of bone marrow-derived cells showed an increased chondrocyte population by incubation periods. There was no significant different (*p* > 0.05) of chondrogenic potency between cells isolated from the iliac crest and fracture site of CPT patients.

**Fig. 4:**
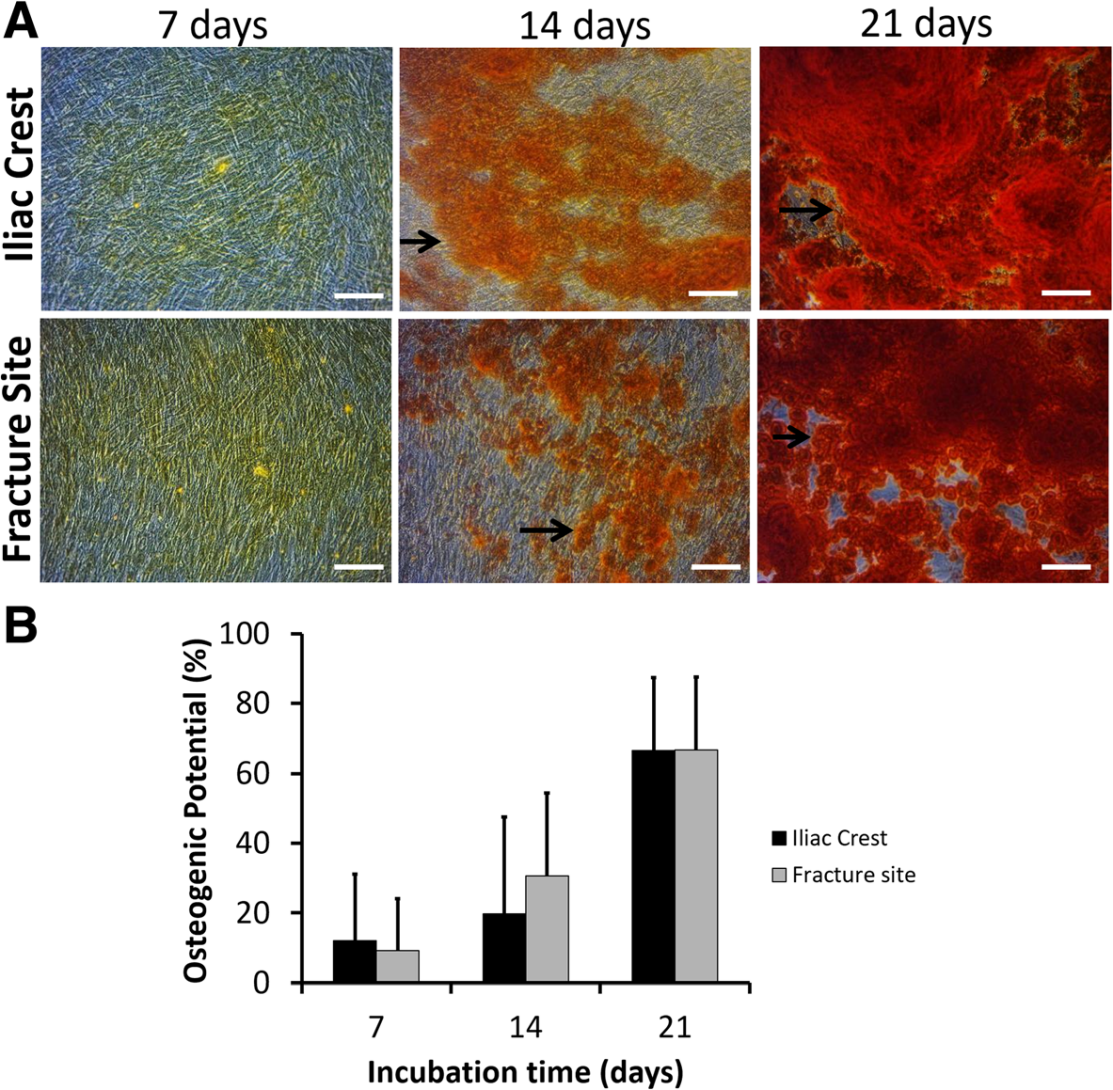
Osteogenic differentiation of MSCs isolated from the iliac crest and fracture site of the tibia from CPT patients. **a** Representative microscopic images of osteogenic assays. Bars and black arrows indicate 100 μm and osteocytes population, respectively. **b** Percentage of osteogenic differentiation potential**.** Data were shown as means, and the vertical bars indicated standard deviations (*n* = 6)

The data tendency from the osteogenic differentiation assay was similar with that from the chondrogenic differentiation assay. Osteocyte population gradually formed after 7 days of incubation. There is no significantly difference (*p* > 0.05) of osteogenic potency between the cells isolated from the iliac crest and fracture site (Fig. 4).

**Fig. 5:**
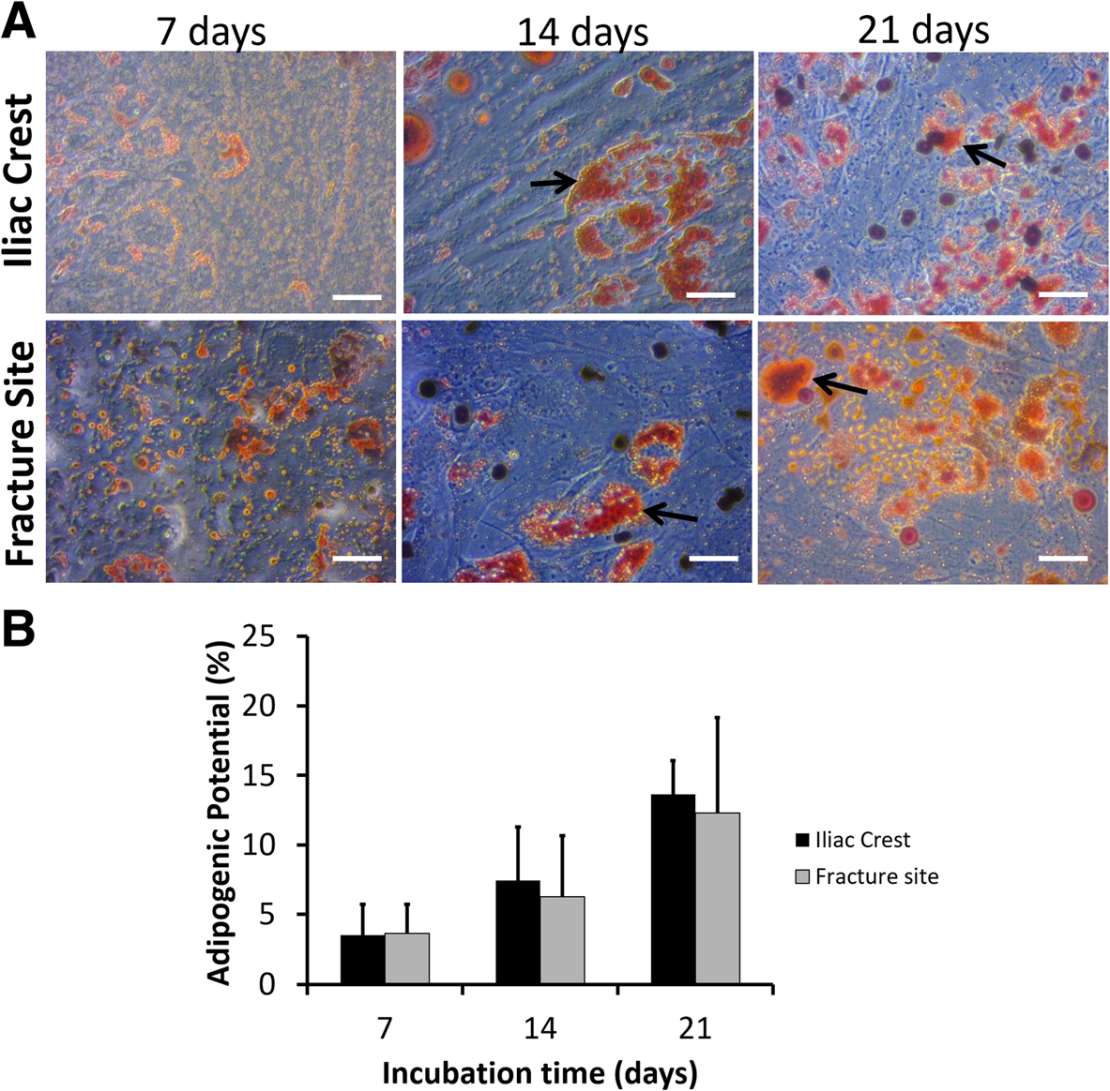
Adipogenic differentiation of MSCs isolated from the iliac crest and fracture site of the tibia from CPT patients. **a** Representative microscopic images of adipogenic assays. Bars and black arrows indicate 100 μm and adipocytes population, respectively. **b** Percentage of adipogenic differentiation potential**.** Data were shown as means and the vertical bars indicated standard deviations (*n* = 6)

Red-stained oil droplets represented the positive result of adipogenic differentiation assay (Fig. 5a). Both of the MSCs were able to differentiate into adipogenic lineage after 7 days of incubation. Moreover, the percentage of adipocyte population became higher after 14 and 21 days of incubation (Fig. 5b). Nevertheless, there was no significant difference (*p* > 0.05) in adipogenic differentiation potency of MSCs from the iliac crest and fracture site of CPT patients.

**Fig. 6:**
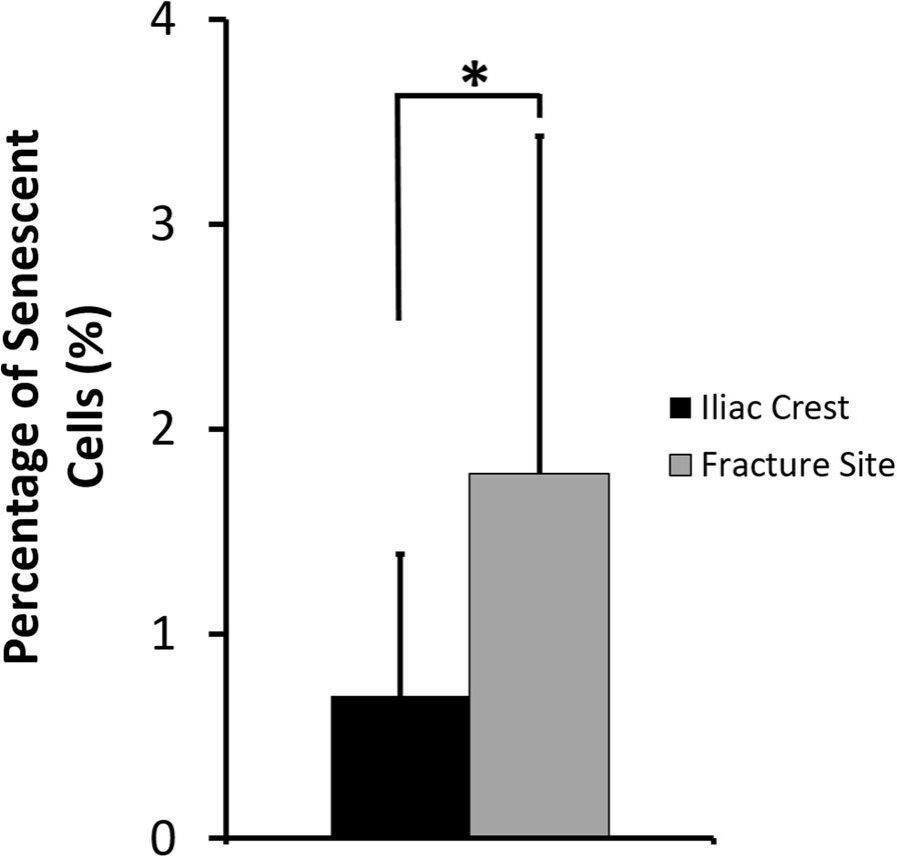
Percentage of senescent cells from the fifth passaged MSCs isolated from CPT patients. Data were shown as means, and the vertical bars indicated standard deviations (*n* = 6). The value of *p* < 0.05 (*) was indicated based on a paired *t* test

Primary cells have a finite proliferation capacity and undergo senescence state after repeated proliferation. On the fifth passage, the percentage of senescence cells of MSCs isolated from the iliac crest and fracture site was assessed. As shown in Fig. 6, there was a tendency for MSCs from the fracture site to have a higher percentage of senescent cells than MSCs from the iliac crest.

**Fig. 7:**
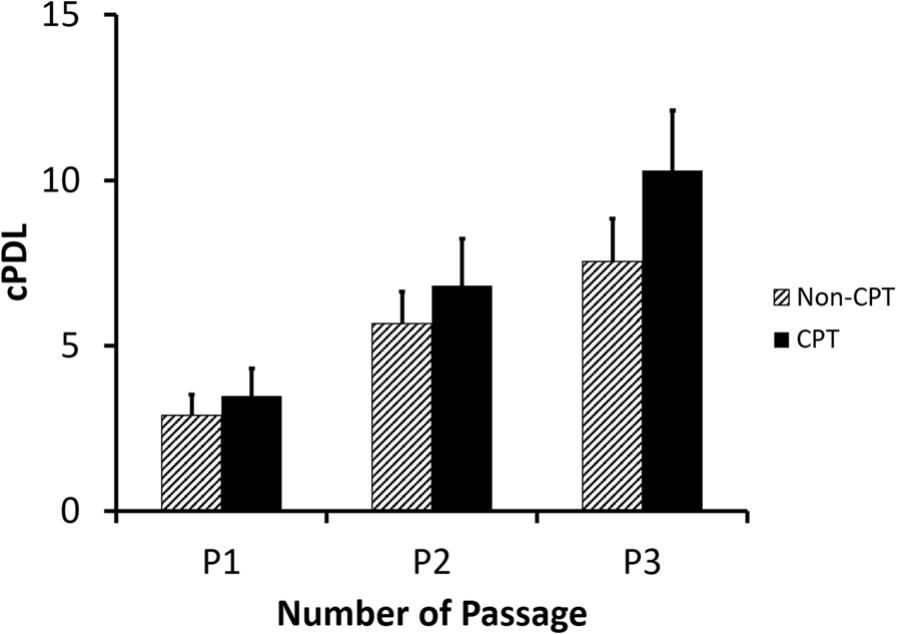
The cPDL of iliac crest-derived MSCs from healthy (non-CPT) and CPT patients. Data were shown as means and the vertical bars indicated standard deviations (*n* = 6)

Figure 7 summarized the cPDL of iliac crest-isolated MSCs from healthy (non-CPT) and CPT patients. The cPDL of CPT patients were slightly higher than those of non-CPT subjects. These result suggested that MSC proliferation from CPT patients were comparable with MSC from healthy subjects.

## Discussion

Treatment for CPT is challenging because patients experience the disorder at a very young age, and success rate of the treatment varies. Surgical treatments for CPT patients frequently involve internal or external fixation with or without bone grafting to improve bone consolidation [19]. Repeated surgical treatments are often needed; however, inevitable outcomes can still occur, i.e., more severe condition or in a worst case lead to amputation [20].

Currently, stem cell therapy offers a regenerative approach to improve the outcome of conventional surgical treatments. MSC therapy has attracted attention due to the potency to improve the surgical methods of CPT treatment by promoting bone and surrounding tissue regeneration. It is likely that autologous MSCs (from a patient’s own body) are more popular than allogeneic MSCs (from a donor). Nevertheless, the studies about MSC quality from CPT patients are still limited.

Bone marrow of the iliac crest is the common source for MSC isolation [21] while a few studies reported a success on isolating MSCs from the bone marrow of the tibia [22]. The number of MSCs in the tibia is typically much lower than that in the iliac crest [22]. In the current study, we evaluated the quality of MSCs from six patients with CPT. MSCs were isolated from the bone marrow of the fracture site of the tibia and iliac crest. The isolated MSCs were cultured on standard culture media with 10% human serum, and passaging was conducted after the cells reached confluence. Experimental data showed that the cPDL increased from the first passage to the fifth passage, suggesting that the cells were actively proliferating. There was no significant difference (*p* > 0.05) in terms of cPDL between MSCs isolated from the iliac crest and those from the fracture site of the tibia in CPT patients.

The MSC specific markers, including CD73, CD90, and CD105 antibodies, were analyzed after the fifth passage. It was clarified that more than 99% of cultured cells were able to express CD73 and CD90 proteins. The CD105^+^ cells were 89% and 86% for MSCs from the iliac crest and fracture site of tibia, respectively. Ng et al. [23] reported the percentage of CD105 cells of human MSCs was around 86% in late passage (sixth passage). The expression of CD105 in human MSCs reduced significantly when human MSCs were cultured in serum-free media; however, the cells could maintain the trilineage potencies [24]. Although CD105 expression is often associated with chondrogenic potential, a recent study clearly stated that enriched CD105 MSCs did not show superior chondrogenic potential [25]. Thus, as long as expressing double-positive CD73/CD90 markers, the cells are potentially classified as MSC. Cells expressing negative markers for MSCs (CD34, CD11b, CD19, CD45, and HLA-DR) in our study were less than 0.5% even after fifth passage.

Surprisingly, percentage of senescent cells from fracture site-derived MSCs was significantly higher than iliac crest-derived MSCs. A study from Bajada et al. [26] investigated the growth potential of MSCs isolated from the fibrocartilaginous tissue at atrophic non-union site. They reported that, in standard culture condition, the proportion of senescent MSCs from this site was higher that MSCs from bone marrow of iliac crest. Their report showed a corresponding agreement with our finding, even though the patient conditions or tissue site were different.

Trilineage potencies of MSCs isolated from CPT patients were evaluated by culturing the cells in specific inducing media. Spontaneous chondrogenic differentiation was detected after culturing the cells over 2 weeks. Similarly, adipogenic differentiation was visually noticed after 2 weeks in adipogenic-stimulating media. In case of osteogenic differentiation, about 10% showed osteogenic potency after a week of induction. The trilineage differentiation potencies increased after longer incubation time. There was no significant difference of trilineage potencies from MSCs isolated from the iliac crest or fracture site of tibia from CPT patients. A case report [27] in a 2-year-old CPT patient suggested that MSCs from his fibrous tissue were able to differentiate into osteogenic, chondrogenic, and adipogenic cells, showing a similar tendency with our finding; the difference was our study used bone marrow-derived MSCs and involved more number of CPT patients.

The cPDL of MSCs from CPT patients was then compared with MSCs from healthy subjects. It was hypothesized that low MSC proliferation might affect the slow bone healing in CPT patients. Surprisingly, the MSC’s cPDL from CPT patients were higher than that of healthy subjects, indicating that CPT-derived MSCs were proliferating in a comparable level with healthy MSCs. In this study, it was difficult to recruit a healthy bone marrow donor with a similar age range with CPT patients. We expected that higher cPDL in CPT patients than in healthy donors was merely caused by age difference.

## Conclusion

The MSC characteristics from the fracture site of the tibia and the iliac crest of CPT patients were similar, in terms of proliferation capacity and trilineage differentiation. It was noticed that the proliferation capacity of iliac crest-derived MSCs from CPT patients was comparable with those from healthy persons. The findings in this study are expected to promote the use of autologous MSC therapy for CPT patients.

## Abbreviations

cPDL: Cumulative population doubling time
CPT: Congenital pseudoarthrosis of the tibia
HBV: Hepatitis B virus
HCV: Hepatitis C virus
HIV: Human immunodeficiency virus
MSC: Mesenchymal stem cell
TORCH: Toxoplasma gondii, other viruses (HIV, measles, and so on), rubella (German measles), cytomegalovirus, and herpes simplex
